# Monitoring the treatment of hepatitis C with directly acting antivirals by serological and molecular methods

**DOI:** 10.1371/journal.pone.0187755

**Published:** 2017-11-10

**Authors:** Elisabetta Loggi, Silvia Galli, Giovanni Vitale, Roberto Di Donato, Ranka Vukotic, Elena Grandini, Marzia Margotti, Valeria Guarneri, Giuliano Furlini, Claudio Galli, Maria Carla Re, Pietro Andreone

**Affiliations:** 1 Dipartimento di Scienze Mediche e Chirurgiche, Centro di Ricerca per lo Studio delle Epatiti, Università degli Studi di Bologna, Bologna, Italy; 2 Unità di Microbiologia, Policlinico S.Orsola-Malpighi, Bologna, Italy; 3 Medical Scientific Liaison, Abbott Diagnostics, Roma, Italy; Kaohsiung Medical University Chung Ho Memorial Hospital, TAIWAN

## Abstract

**Aim:**

To evaluate the potential value of using a serological assay to quantitate the hepatitis C virus core antigen (HCV-Ag) when monitoring patients with chronic hepatitis C being treated with direct-acting antivirals (DAAs).

**Methods:**

Ninety-six patients treated with DAAs, either alone (91) or in combination with PEG interferon (5), were tested for HCV-RNA and for HCV-Ag at baseline and at weeks 2, 4, 8 and 12 during treatment and 12 weeks after completion. The concordance and correlation between the viral parameters as well as the respective kinetics during and after treatment were evaluated.

**Results:**

A sustained viral response (SVR) was achieved in 82 patients (91%), whereas 11 relapsed (R) and 1 showed a virological breakthrough while receiving treatment. HCV-RNA and HCV-Ag showed good concordance (kappa = 0.62) and correlation. No significant differences between SVR and R was observed in either assay at 2 and 4 weeks after the start of treatment. At 8 weeks, HCV-Ag showed higher accuracy than HCV-RNA (AUC: 0.74 vs. 0.55) and there was a significantly greater decrease from baseline in SVR than in R (4.01 vs. 3.36 log10; p<0.05).

**Conclusions:**

Monitoring during treatment with DAAs by using either HCV-RNA or HCV-Ag has only a limited predictive value for SVR. Since those assays are equivalent for identifying a virological relapse, HCV-Ag may be preferred from an economical and organizational perspective.

## Introduction

The treatment of chronic hepatitis C virus infection (CHC) has been revolutionized by the introduction of direct-acting antivirals (DAAs), which are agents that can interfere with different steps of the replicative cycle of a virus [1, 2]. This molecular approach replaces the standard treatment based on a combination of pegylated interferon α (peg-IFNα) and ribavirin (RBV), which acts on several non-specific pathways to boost the antiviral immune response [3]. DAAs lead to viral eradication in more than 90% of patients [2]. In addition to being significantly more effective than interferon-based therapy for curing the infection, these treatments have important additional benefits, including a tolerability profile that makes them suitable for previously excluded patients [4], simplified management due to the shorter treatment duration and an oral route of administration. Unfortunately, the high costs of these therapies currently limit the access to these drugs, thereby requiring strict patient selection and blocking the drugs’ widespread use [5].

Measuring HCV-RNA by using sensitive molecular techniques has been the gold standard for treatment monitoring in the era of IFN-based treatment. Baseline viremia and an early drop in HCV-RNA levels are the strongest elements for predicting the treatment outcome, and viremia measurement during treatment is crucial for establishing the treatment duration and assessing the response-guided therapy [6].

The introduction of DAA regimens has changed virological monitoring because the baseline HCV-RNA levels no longer seem to be response-predictors and because detectable residual HCV-RNA at the end of DAA therapies can occur frequently but does not have an association with subsequent viral relapse [7]. Consistent with this assumption, no futility/stopping rules have been established to date. To simplify treatment monitoring, hepatitis C core antigen (HCV-Ag) is emerging as a new tool for diagnosis and treatment monitoring in CHC.

HCV-Ag is a highly conserved and antigenic protein that is released into the plasma [8, 9] and can be easily quantified due to the availability of an automated platform. HCV-Ag quantification is an indirect measure of viral replication [10, 11], and it has been proven to be useful for treatment monitoring of IFN-based therapy [12, 13] and has even recently been used to monitor DAA-based therapy [14, 15].

In this study, we aimed to assess the accuracy of HCV-Ag for monitoring therapy efficacy compared to RT-PCR in a population of multi-genotype CHC patients who were undergoing treatment with different DAA regimens in a real-life clinical setting.

## Patients and methods

### Patients

Among all the consecutive all-genotype CHC patients who received a DAA-based treatment in between June 2013 and December 2015 at the ITEC Outpatient Clinics of Azienda Ospedaliero-Universitaria di Bologna (Bologna, Italy), 96 CHC patients were selected for the analysis based on the availability of longitudinal serum samples.

The inclusion criteria were adult age (≥18 years), CHC infection confirmed by serum HCV-RNA using an RT-PCR-based method and, in particular, fulfilling the criteria to be eligible for treatment with DAA according to the indications established by the Italian government (Agenzia Italiana del Farmaco-AIFA). The patient population consisted of the following 3 groups:

1. Patients with advanced disease with METAVIR stage F3 (advanced fibrosis) or F4 (cirrhosis);
2. Patients with HCV recurrence after liver transplantation;
3. Patients with extrahepatic manifestations, such as cryoglobulinemia and B-cell lymphoproliferative disease.

The DAA regimen, treatment duration and addition of weight-based ribavirin (RBV) were determined by the treating physician according to current guidelines.

The study was approved by the Ethical Committee of Azienda Ospedaliera di Bologna, (Bologna, Italy), and the patients provided written informed consent.

### Study assessments

SVR was defined as HCV-RNA that was undetectable 12 weeks after completing treatment (SVR12). Post-treatment relapse (R) was defined as confirmed HCV-RNA ≥15 IU/ml during a follow-up in patients with undetectable HCV-RNA at the end of treatment.

Viral breakthrough was defined as a ≥1 log10 IU/ml increase from the nadir of HCV-RNA or when HCV-RNA ≥15 IU/ml after HCV-RNA was undetectable during the treatment.

### HCV-RNA testing and HCV-Ag determinations

The HCV-RNA levels were measured with the COBAS AmpliPrep/COBAS TaqMan HCV Quantitative Test, version 2.0 (Roche; lower limit of quantitation = 15 IU/mL) at baseline as well as after 2, 4, 8, 12, 16 and 24 weeks for patients treated until the 24th week (W2, W4, W8, W12, W16, W24) during treatment. Follow-up measurements were collected at 4 and 12 weeks post-treatment. HCV-RNA was positive if the level was >15 IU/mL, whereas detectable but not measurable HCV-RNA levels were reported as <15 IU/mL and scored as a gray zone. Only samples with an undetectable level of HCV-RNA were considered negative. The HCV genotype and subtype were determined using the Siemens VERSANT HCV Genotype INNO-LiPA 2.0 Assay.

HCV-Ag was retrospectively measured in serum samples obtained at the same time points as the HCV-RNA using an automated chemiluminescent HCV-Ag assay (Abbott Diagnostics, Wiesbaden, Germany) that was performed on the Abbott ARCHITEC T i2000SR platform according to the manufacturer’s instructions. The cut-off value for HCV-Ag detection was 3.0 fmol/L. Levels below 3.0 fmol/L were considered non-reactive, levels between 3 and 10 fmol/L represented the “gray” zone, and levels above 10 fmol/L were positive for HCV-Ag. The dynamic range of the test was 3–20,000 fmol/L, and an automated dilution extended this range to 180,000 fmol/L.

### Statistical analysis

Quantitative variables were expressed as the median and range, and categorical variables were expressed as a number count and proportions. Chi-square or Fisher’s exact test and the Mann-Whitney test were used to compare categorical and continuous variables when appropriate. Correlations were determined with a non-parametric Spearman correlation and kappa statistics. The accuracy of HCV-RNA and HCV-Ag for assessing SVR was evaluated by receiver-operating characteristics (ROC) curves.

A p value of <0.05 was considered statistically significant. All analyses were performed using SPSS for Windows (Statistical Package for the Social Sciences, version 21.0, Armonk, New York, NY, USA), and GraphPad Prism, version 5.

## Results

### Patients and response to treatment

The demographics and clinical and virological features of the enrolled patients are listed in Table 1. The treatment regimens were quite heterogeneous, although most patients were treated with an IFN-free course (91, or 95%), but 5 patients (5%) were treated with a combination of Peg-IFN, Ribavirin and DAA. The duration of treatment was 12 weeks for 65 patients and 24 weeks for 31 patients. Most patients (63%) were cirrhotic, and twelve had received a liver transplantation with HCV recurrence.

**Table 1:** Demographics, clinical and virological features and type of treatment of enrolled patients.

Parameter(s)	Data
Age, years: Median (range)	60.5 (31–86)
Male/Female: N (%)	61/35 (64/36)
Naives/Experienced: N (%)	38/58 (60/40)
Baseline HCV-RNA IU/mL: median (range)	1.2x10^6^ (6.14x10^3^-533x10^6^)
Baseline HCV-Ag fmol/L: median (range)	3,376 (10.79->20,000)
HCV Genotype: N (%)	
1a	11 (11)
1b	60 (62.5)
1 (subtype not available)	1 (1)
2	6 (6)
3	14 (14.5)
4	4 (4)
Treatment duration 12/24 weeks: N (%)	65/31 (68/32)
Anti-HBV core positivity: N (%)	35 (36)
Cirrhosis: N (%)	61 (63%)
Previous liver transplantation: N (%)	12 (12.5)
Treatment regimen: N (%)	
3D ± RBV	16 (17)
SOF ± RBV	23 (24)
SOF + SMV ± RBV	32 (33)
SOF + LDV ± RBV	6 (6)
SOF + DCV	14 (15)
SOF + pegIFNα + RBV	5 (5)

HBV: hepatitis B virus; 3D: Ombitasvir-Paritaprevir-Ritonavir and Dasabuvir; SOF: Sofosbuvir; SMV: simeprevir; LDV: Ledipasvir; DCV: Daclatasvir; pegIFNα: Pegylated Interferon alpha; RBV: Ribavirin.

Overall, SVR12 was achieved in 82 of the 96 patients (85% intention to treat analysis), 11 patients experienced a relapse (11%) and only one showed a breakthrough after 6 weeks from the start of treatment. The remaining 2 patients died due to causes unrelated to liver disease before the completion of the follow-up period, but they were HCV-RNA negative until the last available time point. Relapses were diagnosed in 8 patients after 4 weeks from treatment completion and in 3 patients after 12 weeks. The occurrence of viral relapse was unrelated to the previous treatment (10.5% among treatment-naïve patients, 12.5% among treated patients).

### Baseline HCV-RNA and HCV-Ag values and correlation

All patients were positive for both parameters at the start of treatment. The baseline values of HCV-RNA ranged from 6,14x10^3^ to 533x10^6^ IU/mL (Table 1). The distribution of levels showed that most patients (54/96; 56.3%) displayed levels higher than 1x10^6^ IU/mL, whereas 39 of the 96 patients (40.6%) showed values ranging from 1x10^5^ to 1x10^6^, and the remaining 3 had values lower than 10^5^ IU/mL. The baseline values for HCV-Ag ranged from 10.79 to >20,000 fmol/L (Table 1). The levels distribution showed that most patients (71/96, 74%) displayed levels higher than 1,000 fmol/L, whereas 20 of the 96 patients (20.8%) showed values ranging from 100 to 1,000 and only 5 (5.2%) had values lower than 100 fmol/L. Interestingly, the patients with low HCV-RNA or low HCV-Ag levels at baseline were not the same patients. Neither baseline HCV-RNA nor HCV-Ag levels were different between SVR and non-SVR patients (median HCV-RNA: 1.18x10^6^ vs 2.2 x10^6^ IU/mL p = 0.219; median HCV-Ag: 2,974 vs 4,170 fmol/L, p = 0.358, respectively). The correlation between HCV-RNA and HCV-Ag at baseline was good (Spearman r = 0.767, 95% confidence interval 0.66–0.84, p = 0.000).

### HCV-RNA and HCV-Ag: patterns, concordance and accuracy

**Fig 1:**
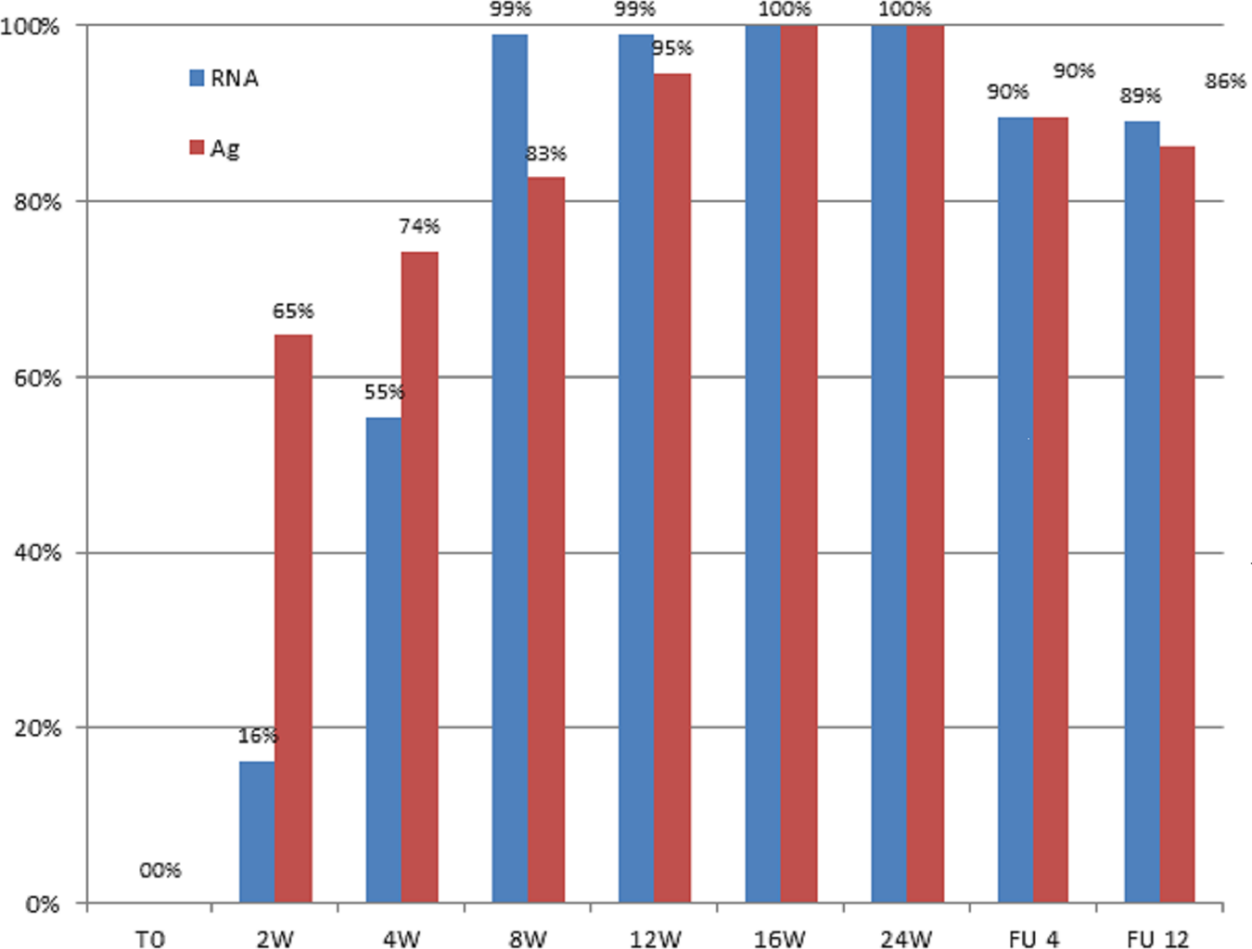
Frequency of negative results for HCV-RNA (blue columns) and HCV-Ag (red columns) during on-treatment monitoring and follow-up of 96 patients treated with DAAs for chronic HCV-infection.

As expected, the decrease in HCV-RNA during treatment was very rapid since the rate of negativity for HCV-RNA went from 16% after 2 weeks to 55% after 4 weeks and 99% after 8 weeks of treatment (Fig 1). The kinetics of HCV-Ag were different because negativity was already seen at 63% after 2 weeks but showed a slower progression during treatment (74% after 4 weeks, 83% after 8 weeks) (Fig 1). At the end of treatment, all patients except the breakthrough case were negative for both HCV-RNA and HCV-Ag. At the time of virological relapse, all 11 patients were positive for both HCV-RNA (range: 5,244 to 1.74x10^7^ IU/mL) and HCV-Ag (range: from 10.13 to >20,000 fmol/L).

**Fig 2:**
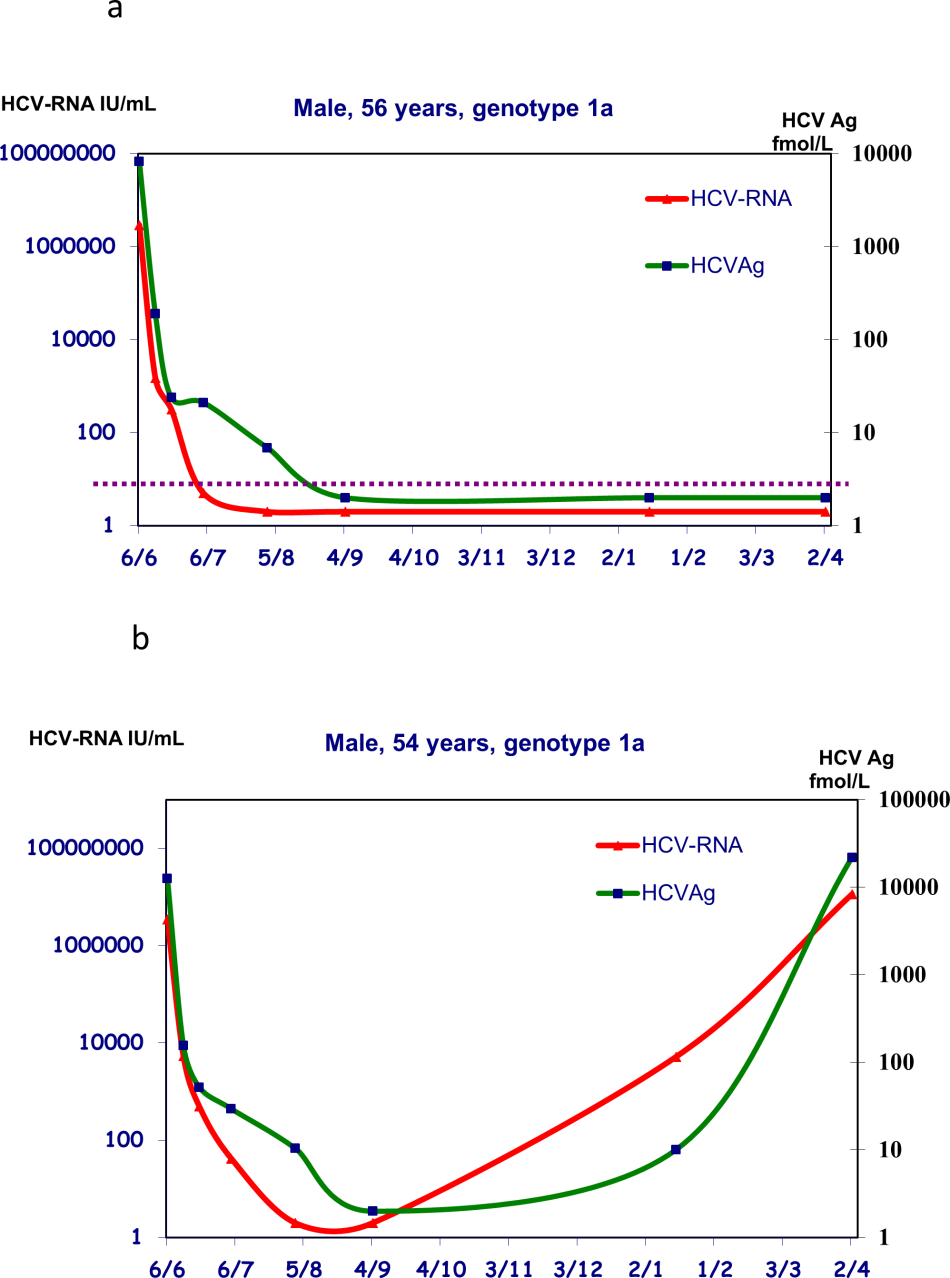
Monitoring profiles for HCV-RNA and HCV-Ag of a representative patient with sustained viral response after 24 weeks of treatment with Sofosbuvir + Ribavirin (2a) and of a patient with relapse after 24 weeks of treatment with Sofosbuvir + Simeprevir + Ribavirin (2b). The horizontal axis reports the dates of blood draws (month, day) from June 2015 to April 2016 after the start of treatment.

Over the complete course of treatment, including baseline and follow-up, a total of 690 samples were assayed for both HCV-RNA and HCV-Ag. Of those, the qualitative concordance between the measures was good (92%; kappa statistics 0.62) (Table 2). Discordant results were observed on both sides, with 39 samples being positive for HCV-RNA and with no detectable HCV-Ag and 13 samples showing opposite outcomes. The latter samples were obtained during treatment and at 4 and 8 weeks after the start of therapy. The median level of HCV-RNA in the 39 samples negative for HCV-Ag was 55 IU/mL with only one specimen (2.6%) yielding a value >1,000 IU/mL. Conversely, the median level of HCV-Ag in the 13 samples negative for HCV-RNA was 15 fmol/L, with only one sample (7.7%) yielding a value >100 fmol/L. Those discrepancies did not account for the different behavior of HCV-RNA and HCV-Ag over the monitoring course because the levels varied with a consistent pattern in SVR and in R (Fig 2). The only difference was a slower decrease in HCV-Ag, which explains how samples were positive for HCV-Ag and had undetectable HCV-RNA.

**Table 2:** Qualitative comparison between HCV-RNA and HCV-Ag on 690 samples from 96 patients treated with DAAs.

Qualitative virological parameters	HCV-RNA positive	HCV-RNA GZ	HCV-RNA negative	Total
HCV-Ag positive	139	5	13	157
HCV-Ag GZ	12	6	25	43
HCV-Ag negative	39	37	414	490
Total	190	48	452	690

The overall agreement (kappa statistic) was good (0.62; 95% confidence limits 0.56–0.68). GZ = gray zone results.

**Fig 3:**
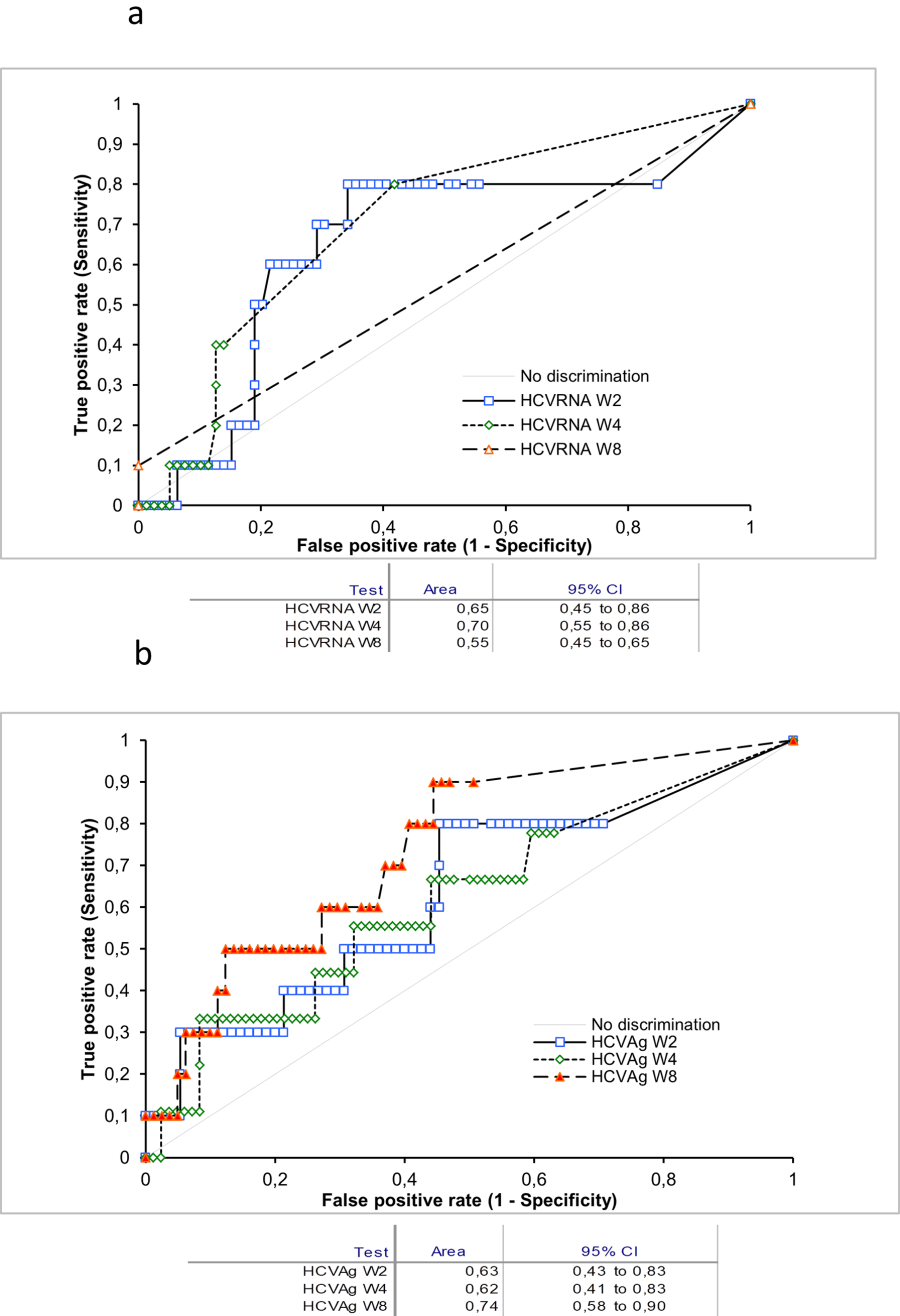
Receiver-operating characteristics (ROC) curves for HCV-RNA (3a) and HCV-Ag (3b) after 2, 4 and 8 weeks after starting treatment with DAAs. The horizontal axis reports the dates of blood draws (month, day) from June 2015 to April 2016 after the start of treatment.

The accuracy of HCV-RNA and HCV-Ag towards SVR was assessed at W2, W4 and W8 during treatment. HCV-RNA levels did not show a strong predictive value for SVR because the area under the ROC curve (AUC) was 0.65 at W2, 0.70 at W4 and 0.55 at W8 (Fig 3A). Indeed, the latter figure is a purely mathematical estimate because all patients with SVR or R did not show detectable viral RNA at that time. HCV-Ag was similarly a poor predictor of SVR at W2 and W4, with AUCs of 0.63 and 0.62, respectively (Fig 3B). However, at W8, the AUC for HCV-Ag was the highest (0.74); this occurred at the same time point when the AUC for HCV-RNA was the lowest, as observed previously. When the kinetics of HCV-RNA and HCV-Ag in the 82 patients infected by HCV genotype 1 (84.5%) were compared to the 14 infected by other genotypes (Table 1), no significant differences were observed.

**Fig 4:**
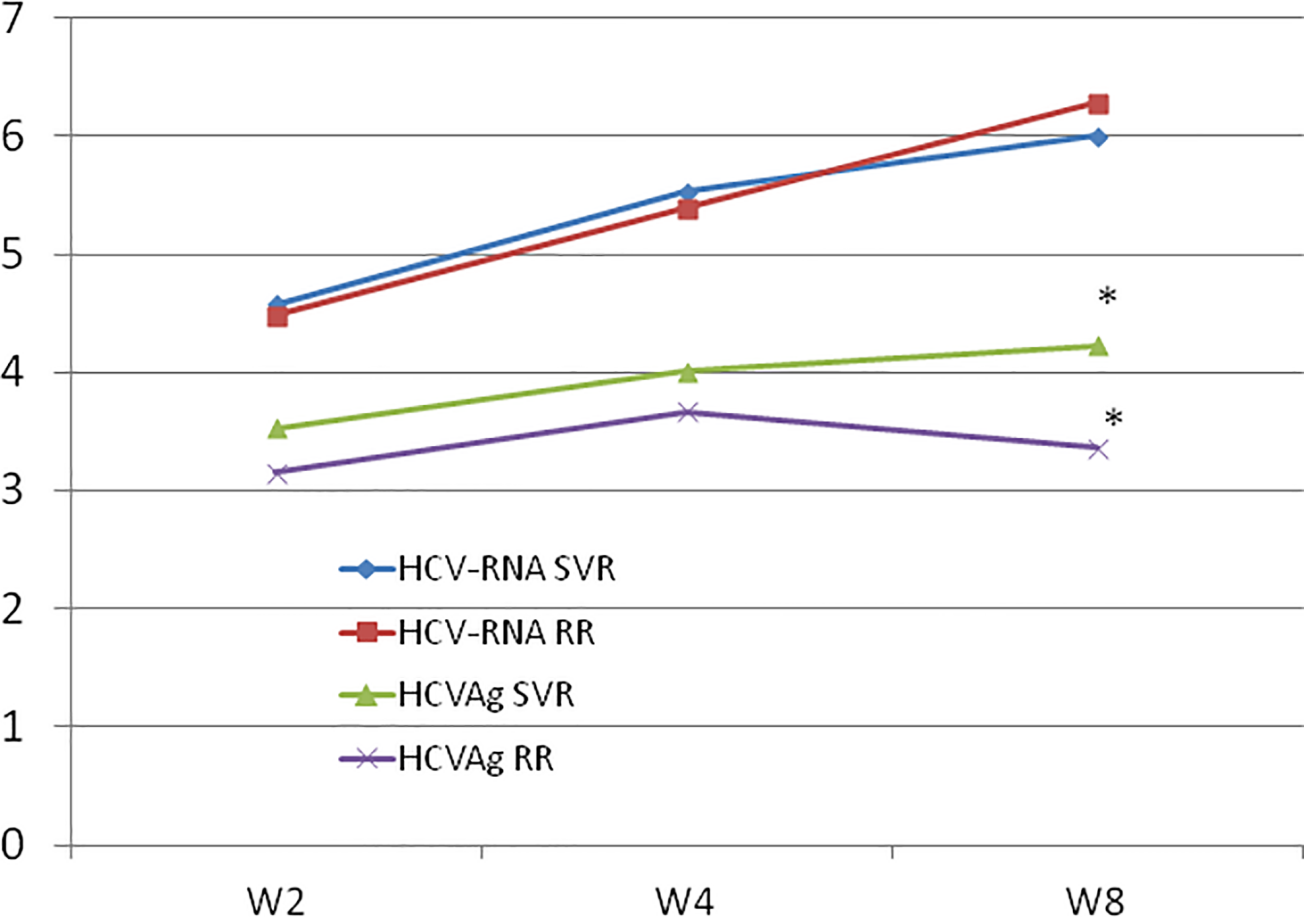
Decrease (logarithmic mean values) from baseline levels of HCV-RNA and HCV-Ag during treatment; SVR = sustained viral response; RR = response with relapse; W2, W4, W8 = weeks 2, 4 and 8 after the start of treatment; * = p<0.05. W = weeks after the start of treatment.

Finally, as a further measure of the efficacy of antiviral treatment, the decrease (expressed in log10 values) in HCV-RNA and HCV-Ag at different time points compared to baseline was calculated (Fig 4). Again, the differences between SVR and R were not significant for HCV-RNA, whereas for HCV-Ag, a significant difference (<0.05) was observed at W8, with a median decrease of 4.23 log10 in SVR compared to 3.36 in R (p<0.05). However, when considering the patients treated for 12 weeks separately from those treated for 24 weeks, this difference was no longer significant.

## Discussion

The outcome of any pharmacological therapy for liver disease due to HCV is still based on testing for HCV-RNA with sensitive techniques because the absence of circulating viral RNA defines both patient responsiveness to drugs and the sustained viral response after treatment. The purpose of HCV-RNA testing may differ according to the type of therapy. Although monitoring patients after 2 and/or 4 weeks of treatment is still recommended for regimens such as interferon in interferon-free DAA schedules, testing after 2/4 weeks is optional and is used to verify adherence [16]. Indeed, negativity for HCV-RNA is obtained early by almost all subjects due to the direct effects of DAAs on viral replication. This conclusion was confirmed in the present study, in which 84% of the patients showed undetectable RNA after 4 weeks and 100% of the patients had the same result after 8 weeks, regardless of the treatment outcome.

However, the same guidelines [16][17] suggest that although doing so provides less strength (A2 level), monitoring may be simplified to increase access to care by skipping assessments during treatment and determining HCV-RNA only at baseline and 12 or 24 weeks after the end of therapy (SVR12 or SVR24, respectively) (A2). In contrast, the most recent guidelines from the American Association for the Study of Liver Disease (AASLD) still recommend quantitative HCV viral load testing after 4 weeks of therapy [17], and the Asian Pacific Association for the Study of the Liver (APASL) guidelines that were issued last year indicate generically that monitoring the HCV loads during treatment is important for response-guided therapy to determine the futility, treatment protocol, and duration [18]. Therefore, a consensus on monitoring for DAA treatment has yet to be reached, and the positive predictive value for SVR of HCV-RNA testing still needs to be ascertained. For this purpose, Maasoumy et al [7] studied the HCV-RNA kinetics from 298 patients with HCV genotypes 1 to 5a who were treated with different Sofosbuvir-based regimens, and they reported that lower levels were attained by SVR patients compared to R in both genotype 1 and genotype 3 infections. For the latter group, the authors indicated that HCV-RNA levels <45 IU/mL or <60 IU/mL (according to the method employed for HCV-RNA quantitation) after 2 weeks of treatment were predictive of treatment outcome and yielded 100% sensitivity. Conversely, the specificity was not outstanding because levels below the proposed thresholds were also attained in 29% and 33% or R, respectively. We were not able to confirm these results, at least for patients with HCV genotype 1 infection (73.5% of our cases), because no significant differences in HCV-RNA levels or decreases could be observed between SVR and R at week 2 or 4 during treatment. This result is relevant because the percentage rates of SVR may currently be lower than the rates reported in the literature, which most likely occurred because of advanced disease and a suboptimal regimen (according to the information available at the time of therapy starting) for some patients (Table 1). In our experience, we observed an 11% rate of R that was unrelated to previous treatment failure and/or other clinical data, and a similar rate (14%) was recently reported by Aghemo et al. [14].

A new approach for DAA monitoring has also been included in the EASL 2017 guidelines. Although the standard methodology is still real-time PCR with high sensitivity (≤15 IU/ml), measuring HCV-Ag has been recommended (A1 level) as an alternative to PCR “when HCV-RNA is not available or not affordable” [16]. HCV-Ag is also recommended in the same guidelines as a way to identify people with ongoing HCV infection (i.e., viral replication) among those individuals who are found to be positive for anti-HCV antibodies. Consistently, the same approach has also been envisioned by the World Health Organization in a very recent document designed to update the diagnostic criteria for both hepatitis B virus and HCV infections [19].

The timing for HCV-Ag testing in treatment monitoring will be the same as HCV-RNA, i.e., at baseline, optional between weeks 2 and 4, at the end of treatment and at the end of post-treatment follow-up [16]. The data reported here are more complete because we could assess both markers of HCV replication at multiple time points during treatment. Overall, the HCV-Ag trends mimicked those of HCV-RNA with different kinetics. The antigen levels decrease more rapidly in the early stages (63% of negativity vs. 16% for RNA) due to the lower analytical sensitivity compared to the real-time PCR for HCV-RNA [10, 11]. The antigen is cleared later, which possibly occurred because the circulating HCV core protein is available not only from virions but also from antigen-antibody complexes [8, 20] that have a longer half-life. When HCV-Ag has been related to treatment outcomes, its overall accuracy was the same as HCV-RNA.

Serological testing for HCV-Ag in patients treated for chronic HCV infection has already been described by several papers, either on patients treated with IFN-based regimens [11–13] or with DAAs [14, 15, 21]. The results of the latter treatments reported high consistency between HCV-RNA and HCV-Ag results both during treatment and at the end of the follow-up period. Our experience complements those studies with more details since we enrolled patients treated with different regimens and infected by different HCV genotypes, and the behavior of the two markers in SVR and in R was assessed separately.

Summarizing this evidence, HCV-RNA and HCV-Ag present similar kinetics in DAA treatment, either during treatment or during follow-up. As expected, HCV-Ag is less sensitive and is often negative in cases with very low levels of HCV-RNA. However, the current schedules do not include stopping rules because all patients are expected to complete the treatment course. Similarly, compared to the Peg-IFN-based treatment, a value for the baseline levels of HCV-RNA is no longer needed in order to tailor therapy, except in select cases [16], so the lower sensitivity of HCV-Ag does not seem to limit the performance of the treatment monitoring. Furthermore, low levels of HCV RNA during treatment or at the end of treatment are not indicative of non-adherence and do not predict a relapse [21, 22], and these are some of the reasons for moving towards a simplified monitoring strategy. Furthermore, the HCV-Ag assay has a very high specificity ranging from 99.8% in the original study [9] to 100% in subsequent observations [10, 19]. However, the potential advantages of using a serological test instead of a virological one is almost clear [9–12, 23]. Cresswell et al [23] demonstrated absolute clinical equivalence and substantial savings for utilizing HCV-Ag in place of HCV-RNA for the diagnosis of acute hepatitis C. Additionally, for treatment monitoring, HCV-Ag allows savings because it is less expensive than RNA [14, 23], does not need specialized technicians and, most of all, has a much faster turnaround time that allows a result to be available in 1 hour compared to up to 7 hours for HCV-RNA [12, 14]. Even though the economic savings may be small compared to the treatment cost, they may be help decrease the overall cost in economically developed countries and will definitely improve access to care for HCV worldwide with the aim of decreasing the prevalence and eventually the social and economic burden of the disease.

A possible limitation of our study is represented by the variety of DAA-based treatment regimens for our patients. Our choice was driven by the objective to report real-life experiences from a reference Hepatology Center, where patients are most frequently referred from other institutions and present with different clinical histories. The rate of virological failure that we observed depends on the heterogeneity of the study population (post-OLT, high percentage of advanced disease) and relies on the fact that a substantial number of patients were treated with a regimen that is no longer considered optimal according to current guidelines. This subset, albeit small, gave us the chance to observe perfect synchronism between viral and serological rebound, which confirms the results from previous studies [14, 21]. The barely significant difference in which there was an HCV-Ag decrease between SR and RR at W8 should not, in our opinion, lead clinicians to check HCV-Ag at this time point because this time point will not represent a stopping rule and will not lead to a change in treatment. However, it is suggested that RNA decay/clearance is linked to the halt in replicative activity due to DAAs action (pharmacological effect), whereas HCV-Ag clearance is linked to the elimination of infected cells that still contain virion components, so a negative HCV-Ag can reflect the complete clearance of infected cells.

Considering that viral relapse was diagnosed equally by HCV-RNA and by HCV-Ag, the latter may represent an alternative clinical value in settings where HCV-RNA is still the preferred or routine method for HCV monitoring.

In conclusion, our study provides further evidence of the clinical equivalency between HCV-RNA and HCV-Ag testing for assessing the response to DAA treatment for chronic HCV infection. A decision on what assay(s) to use in treatment monitoring will rely on the specific setting for the country/region where the therapy is dispensed, including reimbursement policies and continuous interaction and updates between the clinical and microbiology specialists of any given institution.

## Supporting information

S1 Table Study dataset. (XLSX)
